# Diverse models for anti-HIV activity of purine nucleoside analogs

**DOI:** 10.1186/s13065-015-0109-0

**Published:** 2015-05-23

**Authors:** Naveen Khatri, Viney Lather, A K Madan

**Affiliations:** Faculty of Pharmaceutical Sciences, Pt. B. D. Sharma University of Health Sciences, Rohtak, 124001 India; JCDM College of Pharmacy, Barnala Road, Sirsa, 125055 India

**Keywords:** Anti-HIV activity, Superaugmented pendentic topochemical index, Balaban-type index from *Z*-weighted distance matrix, Moving average analysis, Purine nucleoside analogs, Support vector machine

## Abstract

**Background:**

Purine nucleoside analogs (PNAs) constitute an important group of cytotoxic drugs for the treatment of neoplastic and autoimmune diseases. In the present study, classification models have been developed for the prediction of the anti-HIV activity of purine nucleoside analogs.

**Results:**

The topochemical version of superaugmented pendentic index-4 has been proposed and successfully utilized for the development of models. A total of 60 2D and 3D molecular descriptors (MDs) of diverse nature were selected for building the classification models using decision tree (DT), random forest (RF), support vector machine (SVM), and moving average analysis (MAA). The values of most of these descriptors for each of the analogs in the dataset were computed using the Dragon software (version 5.3). An in-house computer program was also employed to calculate additional MDs which were not included in the Dragon software. DT, RF, and SVM correctly classified the analogs into actives and inactives with an accuracy of 89 %, 83 %, and 78 %, respectively. MAA-based models predicted the anti-HIV activity of purine nucleoside analogs with a non-error rate up to 98 %. Therapeutic active spans of the suggested MAA-based models not only showed more potency but also exhibited enhanced safety as revealed by comparatively high values of selectivity index (SI). The statistical importance of the developed models was appraised via intercorrelation analysis, specificity, sensitivity, non-error rate, and Matthews correlation coefficient.

**Conclusions:**

High predictability of the proposed models clearly indicates an immense potential for developing lead molecules for potent but safe anti-HIV purine nucleoside analogs.

## Background

The drug design and development process involves the use of a variety of computational techniques, such as (quantitative) structure-activity relationships [(Q)SAR], molecular mechanics, quantum mechanics, molecular dynamics, and drug-receptor docking [1, 2]. (Q)SAR studies are based on the premise that biological response is a function of the chemical structure [3, 4]. (Q)SAR models reveal a relationship between the structural characteristics of the compounds and their biological activity or environmental behavior [5, 6]. (Q)SAR models predict chemical behavior and simulate adverse effects in laboratory animals, tissues, and cells directly from the chemical structure. This will naturally minimize the need to conduct animal tests so as to comply with the regulatory requirements for human health and eco-toxicology endpoints [7, 8]. The main hypothesis in (Q)SAR is that similar chemicals have similar properties, and even a minor structural change(s) will result in a change in property value(s) [9]. SAR represents classification models that are used when an empirical property is characterized in a (+1/−1) manner, such as soluble/insoluble, active/inactive, toxic/non-toxic, permeable/impermeable, inhibitor/non-inhibitor, ligand/non-ligand, substrate/non-substrate, mutagen/non-mutagen, polar/non-polar, or carcinogen/non-carcinogen [10–15]. *In silico* screening constitutes a vital cost-effective high-throughput process for providing a rapid indication of potential hazards for use in lead prioritization [16].

Machine learning (ML) constitutes a vital area of artificial intelligence (AI) in which models are simply generated by extracting rules and functions from relatively large datasets. ML comprises diverse methods and algorithms such as decision trees, general CHAID models, *k*-nearest neighbors, random forests, Bayesian methods, Gaussian processes, artificial neural networks (ANN), artificial immune systems, kernel algorithms, and support vector machines (SVMs). ML algorithms extract relevant information from empirical dataset through computational/statistical techniques and generate a set of rules, functions, or procedures that allow them to predict the properties of novel objects which have not been included in the learning set. (Q)SAR models derived through ML algorithms are subsequently applied during the drug development process so as to optimize the therapeutic activity, target selectivity, and related physico-chemical and biological properties of the selected molecules [10, 17, 18]. The advantage of AI approaches is that they can be easily applied to learn from examples and to evolve suitable prophesy models in spite of the limited understanding of the underlying molecular processes. The AI approach is also beneficial whenever computational simulations based on fundamental physical models are too expensive to perform [19, 20].

AIDS is one of the most urgent global health problems and is the leading cause of death in Africa and the fourth leading cause of death across the world. Highly active antiretroviral therapy (HAART) has gained considerable success in Western countries. The anti-HIV drug evolution process resembles a crystal ball and involves a plenty of astonishment, expectations, and disappointments. Unfortunately, we continue to be dependent on the predictions of the crystal ball. All of the currently available anti-HIV drugs are far from ideal, and we still face problems of acute and chronic side effects, patient compliance issues, drug resistance, cost, and potency. Hopes of long-term management and eradication depend on increasing available therapeutic options [21, 22].

Purine nucleoside analogs (PNAs) constitute an important group of cytotoxic drugs for the treatment of neoplastic and autoimmune diseases [23]. 9-[4-α-(Hydroxymethyl)cyclopent-2-ene-1-α-yl]guanine (CBV), (−)-β-D-(2R,4R)-1,3-dioxolane-guanosine (DXZ), 3′-azido-3′deoxy-guanosine (AZG), and 2′-C-methylguanosine are all known for their reverse transcriptase inhibiting activity [24]. 3,9-Dihydro-9-dioxo-5H-imidazo(1,2-A) purine nucleosides synthesized from these nucleosides have shown improved anti-HIV activity [25].

In the present study, models of diverse nature have been developed through decision tree (DT), random forest (RF), support vector machine (SVM), and moving average analysis (MAA) using molecular descriptors (MDs) as independent variables for the prediction of the anti-HIV activity of purine nucleoside analogs in human peripheral blood mononuclear (PBM) cells.

## Methods

### Dataset

**Fig. 1:**
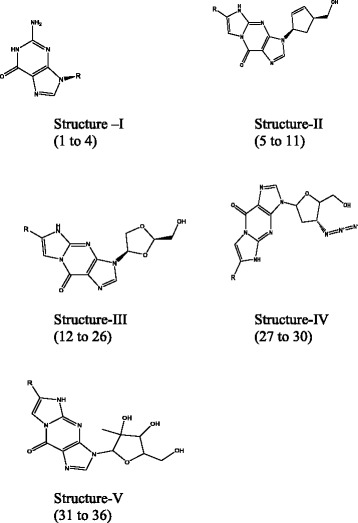
Basic structures of purine nucleoside analogs from serial number 1 to 36 [25]

A dataset comprising 36 purine nucleoside analogs was selected for the present investigation (Fig. 1 and Table 1). The anti-HIV activity of these analogs in human PBM cells has been reported in terms of EC_50_ (effective concentration against 50 % of cell population) by Amblard et al. [25]. The nucleoside analog DXZ possessing an EC_50_ value of 0.51 μM is well known for its anti-HIV activity. DXZ was considered as a reference compound [24]. Accordingly, analogs possessing EC_50_ values of ≤0.51 μM were considered to be active and analogs possessing EC_50_ values of >0.51 μM were considered to be inactive for the purpose of the present study.

**Table 1:** Relationship between molecular descriptors and anti-HIV activity in human PBM cells

Serial number	Basic structure of compound	Substituent (R)	A2	A4	A23	A37	Anti-HIV activity in human PBM cells (EC_50_)				
							Predicted				Reported [25]
							A2	A4	A23	A37	
1	I	3-[4-(Hydroxymethyl)-2-cyclopent-1-yl]	0.919	3.287	9.61	2.16	+	+	±	−	+
2	I	3-(β-D-1,3-Dioxolanyl)	0.922	3.1	9.982	2.254	+	+	±	−	+
3	I	3-(3-Azido-2,3-dideoxy-β-D-erythro-pentofuranosyl)	0.903	4.599	50.599	2.197	+	+	+	−	+
4	I	3-(β-D-2-C-Methyl-ribofuranosyl)	0.848	0.001	615.521	2.332	−	−	−	−	−
5	II	4-MeO-Ph	0.973	11.666	4.866	1.596	−	−	−	−	−
6	II	4-Me-Ph	0.972	11.15	5.338	1.614	−	−	−	−	−
7	II	4-Br-Ph	0.97	11.545	3.883	1.631	−	−	−	−	−
8	II	4-NEt_2_-Ph	0.976	14.051	43.583	1.531	−	−	+	+	+
9	II	4-NMe_2_-Ph	0.976	12.597	43.44	1.573	−	−	+	+	+
10	II	2-Thiophenyl	0.968	9.999	5.937	1.655	−	−	−	−	−
11	II	3-Thiophenyl	0.969	10.637	5.223	1.641	−	−	−	−	−
12	III	Et	0.945	6.157	7.307	1.938	−	−	−	−	−
13	III	Ph	0.971	9.869	2.313	1.674	−	−	−	−	−
14	III	4-MeO-Ph	0.974	11.602	4.849	1.627	−	−	−	−	−
15	III	3-MeO-Ph	0.974	10.545	5.38	1.652	−	−	−	−	−
16	III	2-MeO-Ph	0.97	9.834	6.121	1.678	−	−	−	−	−
17	III	4-Me-Ph	0.973	11.081	5.436	1.648	−	−	−	−	−
18	III	4-Cl-Ph	0.971	11.307	4.712	1.661	−	−	−	−	−
19	III	4-F-Ph	0.971	10.929	5.132	1.655	−	−	−	−	−
20	III	2,4-F-Ph	0.971	10.969	35.94	1.672	−	−	±	−	−
21	III	4-NEt_2_-Ph	0.974	13.458	44.566	1.555	−	−	+	+	+
22	III	4-NMe_2_-Ph	0.976	12.544	44.599	1.601	−	−	+	−	−
23	III	2-Thiophenyl	0.969	9.92	6.049	1.69	−	−	−	−	−
24	III	3-Thiophenyl	0.97	9.951	5.261	1.675	−	−	−	−	−
25	III	4-N_3_-Ph	0.961	12.091	4.532	1.611	−	−	−	−	−
26	III	4-CN-Ph	0.975	12.57	4.893	1.626	−	−	−	−	−
27	IV	Ph	0.939	0.566	6.494	1.645	−	−	−	−	−
28	IV	4-MeO-Ph	0.889	1.923	39.143	1.609	−	−	±	−	−
29	IV	4-NEt_2_-Ph	0.907	4.048	357.424	1.551	+	+	+	+	+
30	IV	4-NMe_2_-Ph	0.901	2.76	344.712	1.589	+	+	+	+	+
31	V	Et	0.878	0.018	993.843	1.986	−	−	−	−	−
32	V	4-MeO-Ph	0.937	0.982	1972.793	1.663	−	−	−	−	−
33	V	4-NEt_2_-Ph	2.345	0.94	16,083.229	1.594	−	−	−	−	−
34	V	4-NMe_2_-Ph	0.983	0.939	12,785.121	1.639	−	−	−	−	−
35	V	2-Thiophenyl	0.123	0.923	2081.689	1.72	−	−	−	−	−
36	V	3-Thiophenyl	0.122	0.923	2059.054	1.706	−	−	−	−	−

+, active; −, inactive; ±, transitional

### Molecular descriptors

The MDs used in the current study include constitutional, physico-chemical, topostructural, topochemical, and topological charge indices, walk and path counts, information-based indices, and a wide variety of 3D descriptors. The majority of 2D and 3D MDs utilized in the present study were calculated using the Dragon software (version 5.3). Most of these MDs are reviewed in the textbook by Todeschini and Consonni [26]. An in-house computer program was also employed to calculate MDs which were not included in the Dragon software. Initially, MDs with significant degenerate values were omitted from the large pool of MDs calculated through both the Dragon software and the in-house computer program. For the remaining MDs, a pairwise correlation analysis was carried out (one of any two indices with *r* ≥ 0.90 was excluded to minimize redundant information). The abovementioned exclusion technique was utilized to decrease the correlation and collinearity between MDs. Finally, 60 MDs, enlisted in Table 2, were short-listed for the development of models.

**Table 2:** List of molecular descriptors

Code	Name of descriptor
A1	Eccentricity index, ECC
A2	Spherocity index, SPH
A3	Molecular connectivity topochemical index, *χ* ^*A*^
A4	Shape profile no. 20, SP20
A5	Shape profile no. 07, SP07
A6	Shape profile no. 08, SP08
A7	Eccentric adjacency topochemical index
A8	Radial distribution function - 10.5/weighted by atomic masses, RDF105m
A9	Second Zagreb index M2, ZM2
A10	Augmented eccentric connectivity topochemical index, ^*A*^ *ξ* ^*c*^
A11	Mean information content on the distance magnitude, IDM
A12	Molecular profile no. 10, DP10
A13	Molecular profile no. 11, DP11
A14	Molecular profile no. 12, DP12
A15	Molecular profile no. 13, DP13
A16	Molecular profile no. 14, DP14
A17	Radius of gyration (mass weighted), RGyr
A18	Eccentric connectivity topochemical index, *ξ* ^*c*^
A19	Connective eccentricity topochemical index, *C* ^*ξ*^
A20	Average vertex distance degree, VDA
A21	Mean square distance index (Balaban), MSD
A22	Schultz molecular topological index, SMTI
A23	Superaugmented pendentic topochemical index-4, ^*SA*^ *∫* ^*P-*4*c*^
A24	Gutman MTI by valence vertex degrees, GMTIV
A25	Xu index, Xu
A26	Mean Wiener index, WA
A27	Superadjacency topochemical index, *∫* ^*AC*^(*G*)
A28	Harary *H* index, Har
A29	Quasi-Wiener index (Kirchhoff number), QW
A30	First Mohar index, TI1
A31	Weiner’s topochemical index, *W*_*c*_
A32	Reciprocal hyper-detour index, Rww
A33	Distance/detour index, D/D
A34	All-path Wiener index, Wap
A35	Superaugmented eccentric connectivity topochemical index-3, ^*SAc*^ *ξ* ^*c*^_3_
A36	Wiener-type index from *Z*-weighted distance matrix (Barysz matrix), WhetZ
A37	Balaban-type index from *Z*-weighted distance matrix (Barysz matrix), JhetZ
A38	Maximal electrotopological negative variation, MAXDN
A39	Molecular electrotopological variation, DELS
A40	Superaugmented eccentric connectivity topochemical index-4, ^*SAc*^ *ξ* ^*c*^_4_
A41	Three-path Kier alpha-modified shape index, S3K
A42	Centralization, CENT
A43	Distance/detour ring index of order 9, D/Dr09
A44	Molecular connectivity index, *χ*
A45	Eigenvalue 11 from edge adjacency matrix weighted by resonance integrals, EEig11r
A46	Average geometric distance degree, AGDD
A47	Absolute eigenvalue sum on geometry matrix, SEig
A48	Eccentric adjacency index, *ξ* ^*Α*^
A49	3D-MoRSE - signal 26/unweighted, Mor26u
A50	3D-MoRSE - signal 25/weighted by atomic Sanderson electronegativities, Mor25e
A51	Augmented eccentric connectivity index, ^*A*^ *ξ* ^*c*^
A52	First component size directional WHIM index/unweighted, L1u
A53	K global shape index/weighted by atomic Sanderson electronegativities, Ke
A54	Superpendentic index, ∫*P*
A55	Mean information content on the leverage magnitude, HIC
A56	*H* total index/weighted by atomic van der Waals volumes, HTv
A57	*R* maximal autocorrelation of lag 1/weighted by atomic Sanderson electronegativities, R1e+
A58	*R* total index/weighted by atomic polarizabilities, RTp
A59	Superaugmented eccentric connectivity index-1, ^*SA*^ *ξ* ^*c*^_1_
A60	Weiner’s index, *W*

Most of the Dragon descriptors are largely defined in ref. [26]

### Statistical methods

#### Decision tree

DT is a common method that provides both classification and predictive functions simultaneously. A single DT was grown for the prediction of anti-HIV activity and to identify the importance of various MDs used for the present study. A cutoff value dividing the compounds of the dataset into active and inactive with regard to anti-HIV activity was assigned to each MD for every compound. Then, a single MD is identified that split the entire training set into two or more homogenous subsets and shows the lowest possible false assignment before being chosen as parent node. The molecules at each parent node are classified, based on the MD value, into two child nodes, and the resulting child nodes or subsets are split into sub-subsets, generally using different MDs. The majority vote of the molecules reaching the same terminal node in the training set decides the prediction for a molecule to reach a given terminal node. In this manner, DT created an interactive branching topology in which the branch taken at each intersection is determined by a rule related to a MD of the molecule, and lastly, each terminating leaf of the tree is assigned to a particular category, i.e., A (active) or B (inactive) [27–30]. In the present study, RPART library was added in R program (version 2.10.1) to grow DT.

#### Random forest

RF is a well-known ensemble of unpruned trees generated through the systematic use of bootstrap samples of the training data for building forests (multiple trees) and random subsets of variables to facilitate the best possible bifurcation at each node [31, 32]. In the present study, the RFs were grown with the R program (version 2.10.1) using the random forest library.

#### Support vector machine

SVM is a relatively new classification technique. SVM involves drawing a boundary between groups of samples that fall into different classes. The SVM methodology comprised reducing the pool of 30 descriptors to a smaller size by eliminating the related variables, followed by development of classification models [33, 34]. Statistica v. 7.0 was used for the generation of SVM models. The classification models were generated using the training set of compounds followed by the validation of the best model using the test set of compounds [35]. Every third compound of the dataset was included in the test set. SVM model validity was also checked by cross-validation, i.e., leave-one-out method. SVM models were also validated by tenfold cross-validation. The kernel type that was adopted in the present work was the polynomial function. The first task was the assignment of each molecule to one class, namely ‘actives’ or ‘inactives’ based on the cutoff value (EC_50_ = 0.51 μM) of the reference compound.

#### Moving average analysis

MAA was utilized so as to facilitate the construction of single MD-based models for predicting the anti-HIV activity of purine nucleoside analogs. For the selection and evaluation of range-specific characteristics, exclusive activity ranges were determined from the frequency distribution of therapeutic response level. This was accomplished by initially plotting the relationship between index values and anti-HIV activity and subsequently identifying the active range by scrutinizing the resultant data by maximization of moving average with regard to active purine nucleoside analogs (<35 % = inactive, 35 % to 65 % = transitional, and >65 % = active) [36]. Biological activity was assigned to each analog involved in the dataset, which was subsequently compared with the reported anti-HIV activity (Table 1). Average values of EC_50_ and selectivity index (SI) were calculated for each range of the proposed models.

#### Model validation

DT-based models were validated using the tenfold cross-validation (CV) method [37]. The performance of the proposed models was evaluated by calculating the overall accuracy of prediction, sensitivity, specificity, non-error rate (arithmetic mean of sensitivity and specificity) [38, 39], and Matthews correlation coefficient (MCC) [40]. MCC is generally regarded as being one of the best statistical techniques which account for both over- and underprediction. MCC takes both sensitivity and specificity into account, and its value ranges from −1 to +1. Higher values of MCC indicate better predictions [41, 42]. The statistical importance of MDs used in building predictive models was also appraised by intercorrelation analysis. The degree of correlation was appraised by Spearman’s rank correlation coefficient ‘*r*’. Pairs of MDs with *r* ≥ 0.97 are considered to be highly inter-correlated while those with 0.68 ≤ *r* ≤ 0.97 to be appreciably correlated; MDs with 0.36 ≤ *r* ≤ 0.67 are weakly correlated whereas the pairs of MDs with low *r* values (<0.35) are not inter-correlated [43, 44].

## Results and discussion

AIDS is the fourth leading cause of death worldwide. Inhibition of the human immunodeficiency virus and sustained suppression of viral replication reduce morbidity and prolong life in patients with HIV infection. This virus is therefore a major target for the structure-based inhibitors design.

Finding that the structure of a molecule has an important role in its therapeutic activity coupled with the need for safer potent drugs to be developed with minimum animal sacrifice, expenditure, and time loss has led to the origin of structure-activity relationship (SAR) studies [45]. The inherent problem in the development of a suitable correlation between chemical structures and biological activity can be attributed to the non-quantitative nature of chemical structures. MDs translate chemical structures into characteristic numerals and facilitate (Q)SAR studies [46, 47].

**Fig. 2:**
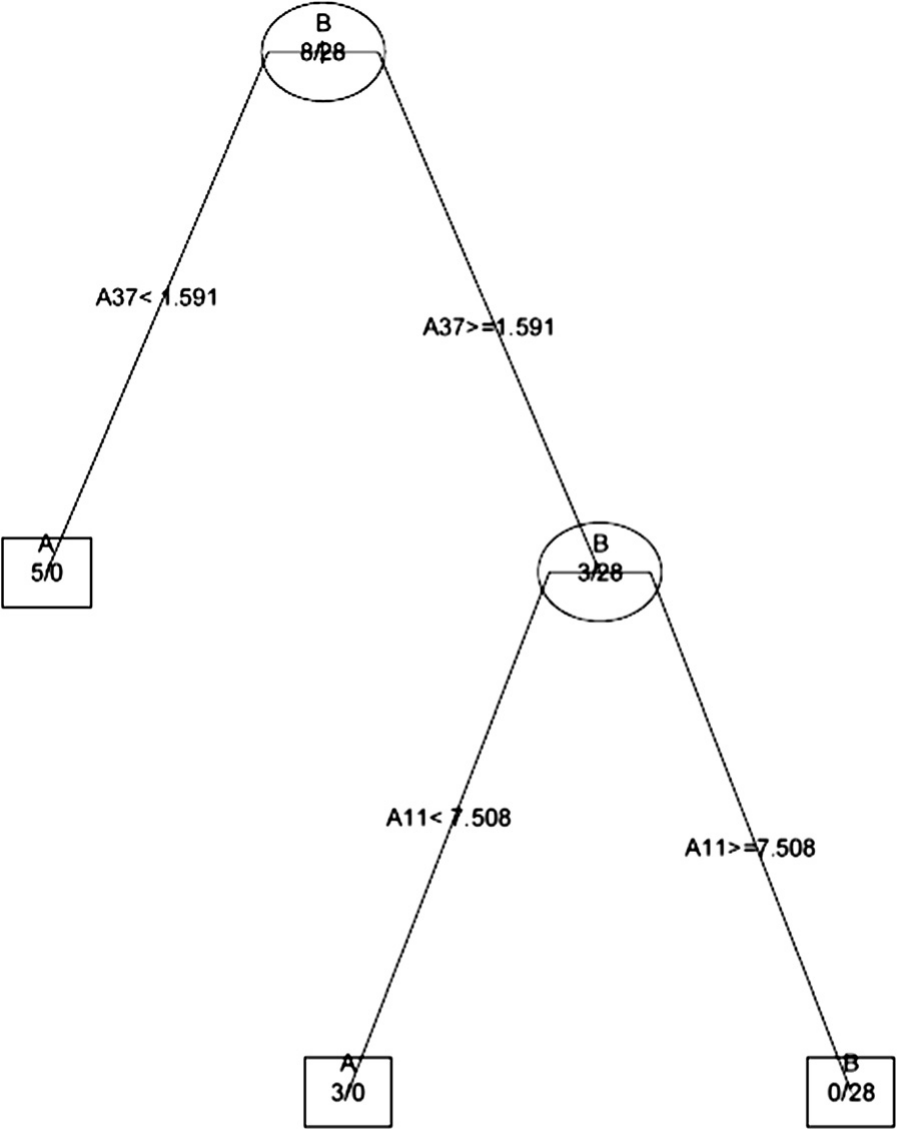
A decision tree for distinguishing active purine nucleoside analogs (A) from inactive analogs (B)

In the present study, the relationship between anti-HIV activity and the structure of purine nucleoside analogs has been investigated and suitable models developed using diverse classification techniques, i.e., DT, RF, SVM, and MAA. DT was built from a set of 60 MDs enlisted in Table 2. The MD at the originating node is the most significant, and the significance of MD decreases with the gradual increase in the tree height [27–30]. The classification of purine nucleoside analogs as inactive and active using a single tree, based on the Balaban-type index from *Z*-weighted distance matrix, A37, *JhetZ index*, and mean information content on the distance magnitude, A11, *IDM index*, has been depicted in Fig. 2. The DT identified the Balaban-type index from *Z*-weighted distance matrix, A37, *JhetZ index*, as the most important index.

A37, i.e., the Balaban-type index from *Z*-weighted distance matrix, *JhetZ index*, is based on the Barysz matrix and was developed by Barysz et al. It may be expressed as per the following:

$$ {J}_{\mathrm{B}}=\frac{q}{\left(\mu +1\right)}{\displaystyle {\sum}_{edges}^G\left(\frac{1}{\sqrt{S_i{S}_j}}\right)} $$

where *S*_*i*_*S*_*j*_ represents the product of the distance sums of the adjacent pairs of vertices *i* and *j* in a graph *G*. The cycloatomic number of the graph is represented by *μ*, and it indicates the number of independent cycles in the graph [48, 49].

The DT classified the analogs with an accuracy of >99.9 % in the training set. The sensitivity, specificity, non-error rate, overall accuracy of prediction, and MCC of the tenfold cross-validated set was of the order of 75 %, 93 %, 84 %, 89 %, and 0.68, respectively (Table 3). A high value of MCC simply indicates the robustness of the proposed DT-based model.

**Table 3:** Confusion matrix for anti-HIV activity of purine nucleoside analogs in human PBM cells

Model	Description	Ranges	Number of compounds predicted		Sensitivity (%)	Specificity (%)	Non-error rate (%)	Overall accuracy of prediction (%)	MCC
			Active	Inactive					
Decision tree	Training set	Active	08	00	100	100	100	>99.9	1.00
		Inactive	00	28					
	Tenfold cross-validated set	Active	06	02	75	93	84	89	0.68
		Inactive	02	26					
Random forest		Active	05	03	62.5	89	75.7	83	0.52
		Inactive	03	25					
Support vector machine	Training set	Active	04	02	66.7	100	83.3	93	0.78
		Inactive	00	21					
	Test set	Active	01	01	50	86	68	78	0.36
		Inactive	01	06					

The recognition rate of decision tree-, random forest-, and support vector machine-based models is also shown

A11, i.e., mean information content on the distance magnitude, *IDM index*, is one of the information indices reported by Bonchev et al. It may be expressed as per the following:

$$ {\overline{\mathrm{I}}}_D^W=-{\displaystyle {\sum}_{n=1}^G\left({k}_n\frac{n}{W}{ \log}_2\frac{n}{W}\right)} $$

where *W* is the Wiener index, *k*_*n*_ is the number of distances of equal *n* value in the triangular submatrix *D*, and *G* is the maximum distance value [50].

The RFs were grown utilizing 60 MDs as enlisted in Table 1. The RF classified purine nucleoside analogs with regard to anti-HIV activity with an accuracy of 83 % and the out-of-bag (OOB) estimate of error was 17 %. The sensitivity, specificity, non-error rate, accuracy of prediction, and MCC value of the RF-based model for the tenfold cross-validated set were found to be 62.5 %, 89 %, 75.7 %, 83 %, and 0.52, respectively (Table 3). A high value of MCC simply indicates the robustness of the proposed RF-based model.

SVM-based classification models were built utilizing a small pool of topological descriptors as specified in the Methods section. The dataset was divided into a training and a test set based on a random test set selection comprising 27 compounds in the training and 9 compounds in the test set, respectively. The models were built using the training set molecules and subsequently validated by test set molecules. The SVM model for the training set resulted in a specificity of 100 % and an accuracy of prediction of 93 %. The sensitivity, specificity, non-error rate, overall accuracy of prediction, and MCC of the test set was of the order of 50 %, 86 %, 68 %, 78 %, and 0.36, respectively (Table 3).

Four single index-based models were developed using MAA (Table 4). The Balaban-type index from *Z*-weighted distance matrix: *index* A37, identified as the most important index by the decision tree, was used to construct a model for the prediction of the anti-HIV activity of purine nucleoside analogs. Three more indices, i.e., spherocity index, SPH, A2; shape profile no. 20, SP20, A4; and superaugmented pendentic topochemical index-4, ^*SA*^*∫*^*P-*4*c*^, A23, were also used to develop the models for predicting the anti-HIV activity of purine nucleoside analogs.

**Table 4:** Proposed MAA models for the prediction of anti-HIV activity of PNAs in human PBM cells

Descriptor	Nature of range in the proposed model	Descriptor value	Number of compounds in each range		Sensitivity (%)	Specificity (%)	Non-error rate (%)	Overall accuracy of prediction (%)	MCC	Average EC_50_ (μM) of correctly predicted compounds in each range	Average SI of correctly predicted compounds in each range
			Total	Correctly predicted							
A2	Lower inactive	<0.901	3	3	63	100	81.5	91.7	0.75	67.1	15.205
	Active	0.901 to 0.922	5	5						0.182	825.741
	Upper inactive	>0.922	28	25						33.613	66.665
A4	Lower inactive	<2.76	9	9	63	100	81.5	91.7	0.75	78.855	6.544
	Active	2.76 to 4.599	5	5						0.182	825.741
	Upper inactive	>4.599	22	19						17.47	43.186
A23	Lower inactive	<9.61	18	18	100	96	98	96.9	0.91	18.056	36.560
	Transitional	9.61 to 43.43	4	NA						4.138	102.32
	Active	43.44 to 357.424	7	6						0.141	859.542
	Upper inactive	>357.424	7	7						100	100
A37	Active	1.531 to 1.589	5	5	63	100	81.5	91.7	0.75	0.134	920.339
	Inactive	>1.589	24	21						37.201	31.408

*NA* not applicable

A2, i.e., spherocity index, SPH, is one of the geometrical descriptors given by Robinson et al. and may be expressed as:

$$ {\varOmega}_{\mathrm{SPH}}=\frac{3{\lambda}_3}{\left({\lambda}_1+{\lambda}_2+{\lambda}_3\right)}\kern2em 1\ge {\varOmega}_{\mathrm{SPH}}\kern0.5em \ge \kern0.5em 0 $$

where *λ*_1_, *λ*_2_, and *λ*_3_ are the eigenvalues of the auto-covarience matrix used in the principal component analysis of the molecule. The Ω_SPH_ value ranges from unity for totally spherical molecules to zero for totally flat molecules [51].

A4, i.e., shape profile no. 20, SP20, is one of the Randic molecular profiles described by Randic and may be expressed as:

$$ S=N+{}^1Rx+{}^2R/2!{x}^2+{\kern0.5em }^3R/3!{x}^3+\kern0.5em {}^4R/4!{x}^4 \dots \kern1em \dots \kern0.5em {}^nR/n!{x}^n $$

$$ S={}^1S,\kern0.5em {}^2S,\kern0.5em {}^3S,\kern0.5em {}^4S \dots \kern1em  \dots {}^nS $$

where *N* is a constant indicating the size of the system. ^1^*R*, ^2^*R*, ^3^*R*… are the averages of the row sums in the ^1^*D*, ^2^*D*, ^3^*D*… matrix, respectively. *D* is the geometry distance matrix of a structure [52].

A23, i.e., superaugmented pendentic topochemical index-4, is the topochemical version of the topological descriptor (superaugmented pendentic index-4) reported by Dureja et al. [53]. Superaugmented pendentic index-4 is expressed as:

$$ {}^{SA}{\displaystyle {\int}^{P-4}}\left({G}_{k,n}\right)={\displaystyle {\sum}_{i=1}^n\frac{p_i{m}_i}{e_i^4}} $$

Superaugmented pendentic topochemical index-4 may be defined as the summation of the quotients of the product of all the non-zero row elements in the chemical pendent matrix and product of chemical adjacent vertex degrees and the fourth power of the chemical eccentricity of the concerned vertex for all vertices in a hydrogen-suppressed chemical molecular graph and may be expressed as:

$$ {}^{SA}{\displaystyle {\int}^{P-4}}{\left({G}_{k,n}\right)}^c={\displaystyle {\sum}_{i=1}^n\frac{p_{ic}{m}_{ic}}{e_{ic}^4}} $$

where *p*_*ic*_ is the chemical pendenticity and is obtained by multiplying all the non-zero row elements in the chemical pendent matrix, *∆Pc*, of a chemical graph (*G*_*k*,_*n*)*c. ∆Pc* is a sub-matrix of the chemical distance matrix and is obtained by retaining the columns corresponding to pendent vertices. *m*_*ic*_ is the augmented chemical adjacency and is defined as the product of chemical degrees of all the vertices *v*_*j*_ adjacent to vertex *v*_*i*_. *e*_*ic*_ is the chemical eccentricity of vertex *v*_*i*_, and *n* is the number of vertices in graph *G* [54–56].

The results of the intercorrelation analysis (Table 5) reveal that the pairs A2:A23 and A23:A37 were not correlated while the pairs A4:A23, A4:A37, and A2:A37 were found to be weakly correlated. The accuracy of prediction for all the four MAA-based models varies from 91.7 to 96.9 %, indicating high predictability (Table 4).

**Table 5:** Intercorrelation matrix

	A2	A4	A23	A37
A2	1.00	0.85	−0.13	−0.62
A4		1.00	−0.39	−0.47
A23			1.00	−0.11
A37				1.00

**Fig. 3:**
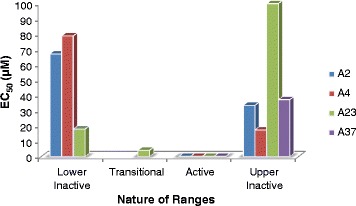
Average EC_50_ of anti-HIV activity of correctly predicted PNAs in various ranges of MAA-based models

The average EC_50_ value of the correctly predicted analogs in the active ranges in MAA-based models varied from 0.134 to 0.182 μM. Such a low average EC_50_ value signifies high potency of the active ranges (Fig. 3).

**Fig. 4:**
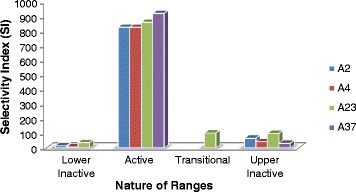
Average SI against PBM cells of correctly predicted PNAs in various ranges of MAA-based models

Drug safety evaluation is the key part of drug discovery and development process to identify those that have an appropriately balanced safety-efficacy profile for a given indication [57]. The therapeutic index (TI), certain safety factor (CSF), protective index (PI), therapeutic window (TW), and selectivity index (SI) are some of such important parameters that can be used to achieve this balance. TI may be defined as the ratio of LD_50_/ED_50_, where LD_50_ is defined as the single dose of a therapeutic agent that can be likely to cause death in 50 % of the animal population and ED_50_ is defined as the single dose of a therapeutic agent that can be likely to cause a particular effect to occur in 50 % of the animal population [58–60]. Similarly, SI is calculated for a drug molecule in the case of cell studies, and it may be defined as the ratio of CC_50_ to EC_50_, where CC_50_ and EC_50_ represent cytotoxic and effective concentrations, respectively. It is an indirect measure of the safety of a drug. A high value of SI simply indicates low toxicity and more safety. A high value of SI is a desirable property for any drug candidate so as to minimize toxicity. Therefore, such safety parameters should be determined in the initial stages of the drug discovery process to avoid much costlier late-stage failures [61]. Active ranges of the proposed MAA-based models exhibited high degree of selectivity towards infected human PBM cells as indicated by a greater value of SI for active ranges compared to inactive ranges (Fig. 4). As a consequence, active ranges identified by MAA models have both the desired requirements of a drug molecule, i.e., high potency and safety. Model validation by confusion matrix shows the sensitivity of the models of the order of 63 % to 100 % (Table 4). High values of MCC simply indicate the robustness of the proposed MAA-based models.

The present modeling studies may be of great utility for providing lead molecules through exploitation of active ranges in single MD-based models. The proposed models are unique and differ widely from conventional QSAR models. Both systems of modeling have their advantages and limitations. In the instant modeling, the system adopted has a distinct advantage of identification of narrow active ranges, which may be erroneously skipped during regression analysis in conventional QSAR. Since the ultimate goal of modeling is to provide lead structures, therefore, active ranges of the proposed models can play a vital role in providing lead structures [62]. Therefore, active ranges of the proposed models can naturally play a vital role in providing lead structures.

## Conclusions

Diverse techniques such as DT, RF, SVM, and MAA were successfully used to develop models for anti-HIV purine nucleoside analogs. Models based on DT, RF, and SVM statistical approaches show an accuracy of prediction up to the order of 89 %. The overall accuracy of prediction of MAA-based models varies from 91.7 % to 96.9 % with regard to the anti-HIV activity of purine nucleoside analogs in human PBM cells. High values of sensitivity, specificity, and MCC indicate the robustness of the proposed models. Good predictability, high potency, and safety of the active ranges in the proposed MAA-based models will naturally provide ease in furnishing lead structures for the development of potent but safe anti-HIV purine nucleoside analogs.
